# Safety and efficacy of extracorporeal shock wave lithotripsy vs. flexible ureteroscopy in the treatment of urinary calculi: A systematic review and meta-analysis

**DOI:** 10.3389/fsurg.2022.925481

**Published:** 2022-11-07

**Authors:** Guangda Lv, Wenqiang Qi, Han Gao, Yongheng Zhou, Minglei Zhong, Kai Wang, Yunxing Liu, Qiang Zhang, Changkuo Zhou, Yan Li, Lingling Zhang, Dongqing Zhang

**Affiliations:** ^1^ Department of Urology, Qilu Hospital of Shandong University, Jinan, China; ^2^ Department of Urology, The People’s Hospital of Xin Tai City, Xintai, China; ^3^ Department of Nursing, Qilu Hospital of Shandong University, Jinan, China

**Keywords:** extracorporeal shock wave lithotripsy, flexible ureteroscopy (f-URS), urinary calculi, systematic review, meta-analysis

## Abstract

**Objective:**

This study aims to compare the safety and efficacy of extracorporeal shock wave lithotripsy (SWL) and flexible ureteroscopy lithotripsy (f-URS) in treating urinary tract stones.

**Methods:**

We systematically searched PubMed, Embase, and Cochrane for literature comparing SWL with f-URS. The primary outcomes we focused on were stone-free rate (SFR) and complications; the secondary outcomes were operation time, hospital stay, retreatment rate, number of sessions, and auxiliary procedures rate. We used ReviewManager version 5.4.1 and STATA version 14.2 for meta-analysis.

**Results:**

Seventeen studies with a total of 2,265 patients were included in the meta-analysis, including 1,038 patients in the SWL group and 1,227 patients in the f-URS group. The meta-analysis indicated that patients in the f-URS group had higher SFR than those in the SWL group [odds ratio (OR): 2.00, 95% confidence interval (CI): 1.29–3.12, *p* = 0.002]. In addition, we found no significant difference in complications (OR: 1.08, 95% CI: 0.85–1.37) between the two treatments. Also, we found that the retreatment rate and the auxiliary procedure rate in the f-URS group were significantly lower than those in the SWL group (OR: 0.08, 95% CI: 0.02–0.24, *p* < 0.00001; OR: 0.30, 95% CI: 0.11–0.83, *p* = 0.02). Moreover, the number of sessions in the f-URS group was significantly lower than that in the SWL group [mean difference (MD): −1.96, 95% CI: −1.55 to −0.33, *p* = 0.003]. However, the operation time and hospital stay in the f-URS group were significantly longer than those in the SWL group (MD: 11.24, 95% CI: 3.51–18.56, *p* = 0.004; MD: 1.14, 95% CI: 0.85–1.42, *p* < 0.00001).

**Conclusion:**

For 1–2-cm urinary stones, f-URS can achieve a higher SFR than SWL while having a lower retreatment rate, number of sessions, and auxiliary procedure rate. For urinary stones <1 cm, there was no significant difference in SFR between SWL and f-URS groups. The SWL group has a shorter operative time and hospital stay than the f-URS group.

## Introduction

Urinary calculus is one of the most common and painful diseases in urology (1). Global warming, high-salt diet, urinary tract infection, genetic factors, and so on are common etiological factors (2). Severe urolithiasis may lead to a lot of damage to patients, such as infections and chronic kidney failure (3). Although stones can be present throughout the entire urinary tract, the most common site is the kidney (4). Kidney stones easily descend to the ureter causing severe pain (5).

Extracorporeal shock wave lithotripsy (SWL) and flexible ureteroscopy (f-URS) are the two most common treatments for kidney and upper ureteral stones smaller than 2 cm in diameter (6). SWL is a noninvasive and anesthesia-free procedure with a stone-free rate (SFR) of around 80%. However, the problems such as high retreatment rate and possible kidney damage cannot be ignored. f-URS is an invasive treatment and requires anesthesia assistance. With the development of endoscopy technology, the quality of f-URS to explore the upper urinary tract has greatly been improved. Experiences showed that f-URS might have a higher SFR and a lower risk of kidney damage and bleeding (7, 8). However, higher medical costs, greater surgical difficulty, and greater risk of ureteral injury limit surgeons’ and patients’ preference to use it for treatment (9). At present, guidelines no longer consider SWL as the mandatory first choice for the treatment of stones ≤2 cm. The prospect of using f-URS to treat stones ≤2 cm is promising.

The best treatment for kidney and upper ureteral stones ≤2 cm in diameter is still controversial. Thus, the purpose of this study is to compare the efficacy and safety of SWL and f-URS for the treatment of this kind of stones.

## Methods

We conducted and reported this systematic review and meta-analysis based on the Meta-Analysis of Observational Studies in Epidemiology (MOOSE) guidelines and the PRISMA statement (10, 11). Our study has been registered at the International Platform of Registered Systematic Review and Meta-analysis Protocols (INPLASY; https://inplasy.com) under registration number 202240120.

### Search strategy

All relevant literature on PubMed, Embase, and Cochrane library database were reviewed. The search strategy design included studies comparing SWL and f-URS in treating renal stones ≤2 cm or upper ureteral stones ≤2 cm. The keywords used for the search were “Ureteroscopy,” “Lithotripsy,” “Extracorporeal Shockwave Lithotripsy,” “Calculi,” and “Stone.” The detailed search strategies can be found in the Supplementary material. In addition, we manually searched the reference list of excluded publications to identify any further potential studies.

### Selection criteria

Studies meeting the following criteria were included in this review: (1) studies comparing SWL and f-URS in the treatment of patients with calculi; (2) reported outcomes we were interested in SFR, operation time, complication rate, hospital stay, auxiliary procedure rate, and retreatment rate; (3) stones were less than 2 cm in the diameter; and (4) the age of patients were above 18 years.

The exclusion criteria are as follows: (1) case reports, reviews, conference abstracts, and other ineligible article types; (2) outcomes do not contain the contents of section “Effect of treatments”; and (3) not in English.

### Data extraction

Two reviewers (GL and WQ) independently assessed all eligible studies. Any discrepancies were resolved by discussion with the third reviewer (HG). Each reviewer independently used well-structured and standardized proformas to extract data from all studies included in our review. The following information was extracted from each study: first author's name, year of publication, study design, stone diameter, stone location, detection of stone, evaluation of the treatments, study population, baseline demographic characteristics, and postoperative outcomes (SFR, complication rate, operation time, hospital stay, auxiliary procedure rate, and retreatment rate).

### Outcomes

The main outcomes are SFR and complication rate. The secondary outcomes are operation time, hospital stay, number of sessions, auxiliary procedure rate, and retreatment rate.

### Study quality assessment

We used the Cochrane Collaboration's Tool (version 5.3, The Nordic Cochrane Centre, The Cochrane Collaboration, United States) to evaluate the methodological quality of each randomized controlled trial (RCT) (12). Deviation risks were identified from seven aspects using this tool. The Newcastle–Ottawa Quality Assessment Scale (NOS) was used to evaluate the methodological quality of each included cohort studies (13). Studies with a score ≥6 were eligible for our meta-analysis.

### Statistical analysis

We used odds ratio (OR) and 95% confidence interval (95% CI) to summarize the dichotomous variables, and we used mean difference (MD) and 95% CI to summarize continuous variables, which were presented as mean values with standard deviations (SDs).We did not incorporate the data of studies presenting continuous variables as means and range in the meta-analysis (12).

The Cochrane *Q* test and *I*^2^ statistics were used to quantify the degree of heterogeneity. *I*^2^ values of 25%, 50%, and 75% represent low, moderate, and substantial heterogeneity, respectively (14). A two-sided *p*-value of less than 0.05 was considered statistically significant. We used the random effects model to estimate pooled effect sizes to reduce possible deviations. Egger's test was used to detect potential publication bias in meta-analyses because it is more sensitive. Publication bias testing is not required when the number of included studies is <10. If Egger's *p* value is <0.05, there is substantial publication bias in meta-analyses (15).

We conducted sensitivity analysis by omitting studies one by one to examine the stability of pooled estimates. If there was no significant difference between the adjusted and primary results, our meta-analysis was stable (12).

To compare the efficacy of SWL and f-URS for stones <1 cm and stones of 1–2 cm, respectively, we performed meta-analyses on these two subgroups. If a study only described stones ≤2 cm, the study would not be included in either of the two subgroups. In addition to comparing the different grades of postoperative complications of the two treatments, we performed a meta-analysis on four subgroups. The complication grade was determined according to the Clavien–Dindo classification (16).

All data analysis was performed with ReviewManager software (RevMan version 5.3, The Nordic Cochrane Centre, Cochrane Collaboration, 2014) and STATA (version 14; StataCorp LLC, Texas A&M University, College Station, TX, United States).

## Results

### Literature search

**Figure 1:**
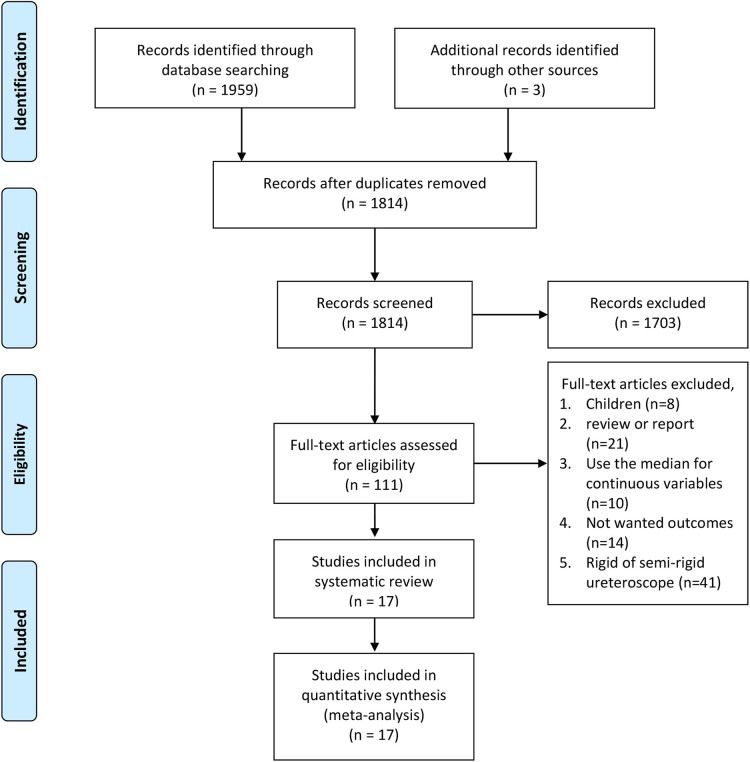
PRISMA flow diagram of literature retrieval. PRISMA, Preferred Reporting Items for Systematic Reviews and Meta-Analyses.

A flow diagram outlining the literature search is shown in Figure 1. Our initial search identified 1,962 records. After checking for duplications and reviewing titles, abstracts, and full texts, we included 17 eligible articles (4, 8, 17–31) in the meta-analysis.

### Information of included studies and patients

The baseline characteristics of the included patients are shown in Table 1. A total of 2,265 patients were finally enrolled in our meta-analysis, of which 1,227 (54.17%) patients were enrolled in the f-URS group and the other 1,038 (45.83%) patients were enrolled in the SWL group. There were eight RCTs (18, 22, 23, 26–29, 31) and nine cohort studies (4, 8, 17, 19–21, 24, 25, 30) included in the meta-analysis.

**Table 1:** Baseline characteristics of the included patients.

Study	Country	Study design	Stone size (cm)	Stone location	Detection of stone	Evaluation of SFR	Endpoint of SFR	Age (year, *X* ± SD)		Gender (female/male)		BMI (kg/m^2^, *X* ± SD)		Stone size (cm, *X* ± SD)	
								f-URS	SWL	f-URS	SWL	f-URS	SWL	f-URS	SWL
Ahmed R (2012)	Egypt	RECT	1–2	Lower pole stone	Ultrasound, KUB	CT	3 months	47.8 ± 10.7	45.4 ± 11.3	11/26	21/41	26.2 ± 6.8	28.2 ± 4.3	13.1 ± 2.4	13 ± 2.3
Anup Kumar (2015)	India	RCT	1–2	Lower calyceal renal calculi	Ultrasound, KUB, CT	Ultrasound, KUB, CT	3 weeks	33.4 ± 1.4	33.1 ± 1.3	23/20	21/21	23.6 ± 1.1	23.4 ± 1.2	13.1 ± 1.1	13.2 ± 1.2
Anup Kumar (2015)	India	RCT	<2	Lower calyceal stones	Ultrasound, KUB, CT	Ultrasound, KUB, CT	3 weeks	35.6 ± 2.1	37.7 ± 2.4	44/46	46/44	NA	NA	12.3 ± 1.1	12.1 ± 1.2
Bhupendra Pal Singh (2014)	India	RCT	1–2	IC stones	Ultrasound, KUB, IVU, CT (when required)	Ultrasound, KUB	1 month	37.65 ± 11.8	34.5 ± 13.07	13/22	15/20	23.45 ± 3.3	22.7 ± 4.35	15.05 ± 3.56	16.45 ± 2.28
Faruk Ozgor (2018)	Turkey	RECT	1–2	Lower pole stones	IVU or CT	CT, KUB	3 months	45.9 ± 14.7	48.6 ± 14.9	65/63	48/65	26.3 ± 5.1	25.6 ± 5.2	12.1 ± 5	11.3 ± 3.1
G. Bozzini (2017)	Germany	RCT	1–2	A single lower pole stone	KUB or CT	KUB at day 10 and a CT scan after 3 months	10 days and 3 months	55.8 ± 16.1	53.3 ± 14.8	106/101	97/97	NA	NA	14.82 ± 2.7	13.78 ± 3.1
H. Aboutaleb (2012)	Egypt	RECT	1–2	Lower calyceal stones	KUB	KUB	2 weeks and 3 months.	47.2 ± 15.2	53.2 ± 19	6/7	5/19	NA	NA	14.5 ± 3.2	15.6 ± 4.3
Ibrahim Kartal (2020)	Turkey	RECT	1–2	Proximal ureteral stones	KUB, ultrasound, and/or CT	KUB, ultrasound, and/or CT	15 days and 3 months	44.5 ± 13.1	43.6 ± 12.6	49/152	35/127	25.3 ± 2.7	24.8 ± 2.1	13.6 ± 2.4	13.4 ± 2.6
Luke H. Chan (2017)	Scotland	RECT	1–2	Lower pole stones	KUB, ultrasound or CT	KUB, ultrasound or CT	1 and 3 months	62.2 ± 15	54.1 ± 13.3	10/11	54/144	NA	NA	13.1 ± 3.7	12.4 ± 2.4
Nevzat Can Sener (2014)	Germany	RCT	<1	Lower pole stones	KUB, ultrasound IVU、CT	KUB, ultrasound IVU, CT	3 months	45.4 ± 6.4	42.9 ± 5.6	29/41	39/31	NA	NA	7.8 ± 1.3	8.2 ± 1.2
Nevzat Can Sener (2015)	United States	RCT	<1	Lower pole stones	KUB, IVU and CT	KUB, IVU, and CT	3 months	36.84 ± 11.7	34.5 ± 11.04	15/35	13/37	NA	NA	8.2 ± 1.2	7.9 ± 1.1
Okan Bas (2014)	Germany	RECT	1–2	A single renal pelvis opaque stone	KUB	KUB	1 month	47.2 ± 14.2	46.4 ± 15.1	17/30	24/28	NA	NA	14.8 ± 2.3	15.3 ± 2.1
Pearle, Margaret S (2008)	United States	RCT	<1	Lower pole stones	KUB, CT	KUB, CT	3 months	49.3 ± 14.2	52.5 ± 12.3	18/17	13/19	28.1 ± 6.6	26.9 ± 8.1	NA	NA
R.M. Vilches (2015)	Chile	RCT	<2	Lower pole stones	fluoroscopy	CT	2 months	43.7 ± 9.2	45.6 ± 13.7	9/15	13/18	26.6 ± 1.5	27.7 ± 3.1	9.7 ± 0.5	9.6 ± 0.6
Ufuk Ozturk (2013)	Turkey	RECT	1–2	Lower pole stones	KUB, ultrasound	KUB	3 months	52	44.2	16/22	98/123	NA	NA	17.3 ± 1.45	17 ± 1.55
Vincent Koo (2011)	UK	RECT	<2	Lower pole r calculi	Ultrasound/CT and/or KUB	Ultrasound/CT and/or KUB	1 month	56.6 ± 15.9	51.2 ± 14.9	15/22	16/35	NA	NA	8.4 ± 2.5	8.5 ± 3.5
Volkmar Tauber (2015)	Austria	RECT	<2	Renal and ureter	KUB or CT	KUB or CT	2–3 weeks, another 6–12 weeks	NA	NA	60/101	60/105	NA	NA	NA	NA

RCT, randomized controlled trial; RECT, retrospective case trial; KUB, radiography of kidneys, ureter, and bladder; CT, computed tomography; BMI, body mass index; f-URS, flexible ureteroscopy; SWL, extracorporeal shock wave lithotripsy; NA, not available; IC, inferior calyceal; SFR, stone-free rate; IVU, intravenous urography.

### Results of studies’ quality assessment

**Figure 2:**
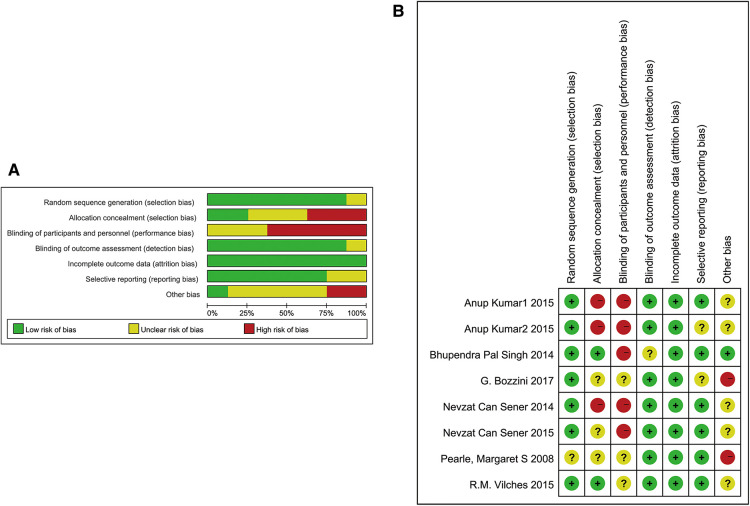
Quality assessment of the RCTs. (**A**) Results of quality assessment summary. (**B**) The quality assessment of each study.

The results of the quality assessment of RCTs are shown in Figure 2. All except one study of RCTs described specific randomization methods. Only one study described allocation concealment, and no study was double-blinded. Except for one study, others mentioned the blinding of outcome assessment. All studies reported complete outcome data. Six articles mentioned that there was no selective reporting. Other biases were low in one study, high in two studies, and unclear in others. Quality assessment of the cohort studies is presented in Table 2. The NOS scores were greater than or equal to 6 in all studies. There were no other risks of bias identified.

**Table 2:** Detailed quality assessment of cohort study.

Items of NOS	Studies								
	Ahmed R	Faruk Ozgor	H. Aboutaleb	Ibrahim Kartal	Luke H. Chan	Okan Bas	Ufuk Ozturk	Vincent Koo	Volkmar Tauber
Selection									
Representativeness of the exposed cohort	*		*	*	*		*	*	*
Selection of the nonexposed cohort	*	*	*	*		*	*	*	
Ascertainment of exposure		*			*		*		*
Demonstration that outcome of interest was not present at the start of the study	*	*	*	*		*		*	*
Comparability									
Comparability of cohorts on basis of the design or analysis	**	*	*	*	*	*	*	*	
Total	8	6	7	6	6	6	7	6	6

NOS, Newcastle–Ottawa Quality Assessment Scale. One * means one point.

### Effect of treatments

#### Stone-free rate

**Figure 3:**
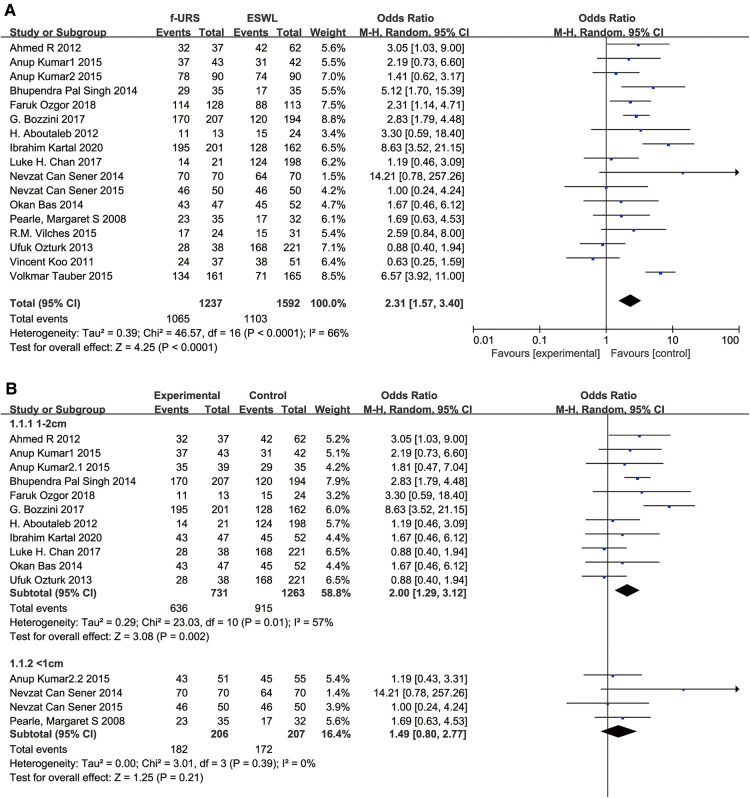
Forest plot and meta-analysis of stone-free rate for (**A**) all studies and (**B**) subgroups of stone length 1–2 cm and <1 cm for f-URS and SWL.

All 17 studies reported SFR in the f-URS group and SWL group. The pooled OR of all 17 studies was 2.31 (95% CI: 1.57–3.40; *p* < 0.0001), indicating a significantly higher SFR in the f-URS group than that in the SWL group. However, significant heterogeneity was found (*I*^2 ^= 66%; *p* < 0.0001). In the stone 1–2 cm subgroup, we found that SFR was higher in the F-URS group than that in the SWL group (OR: 2.00, 95% CI: 1.29–3.12, *p* = 0.002). However, in the stone <1 cm subgroup, no significant difference was found between the two treatment groups (OR: 1.49, 95% CI: 0.80–2.77), as shown in Figure 3. No publication bias was found using Egger's test (*p* = 0.419).

**Figure 4:**
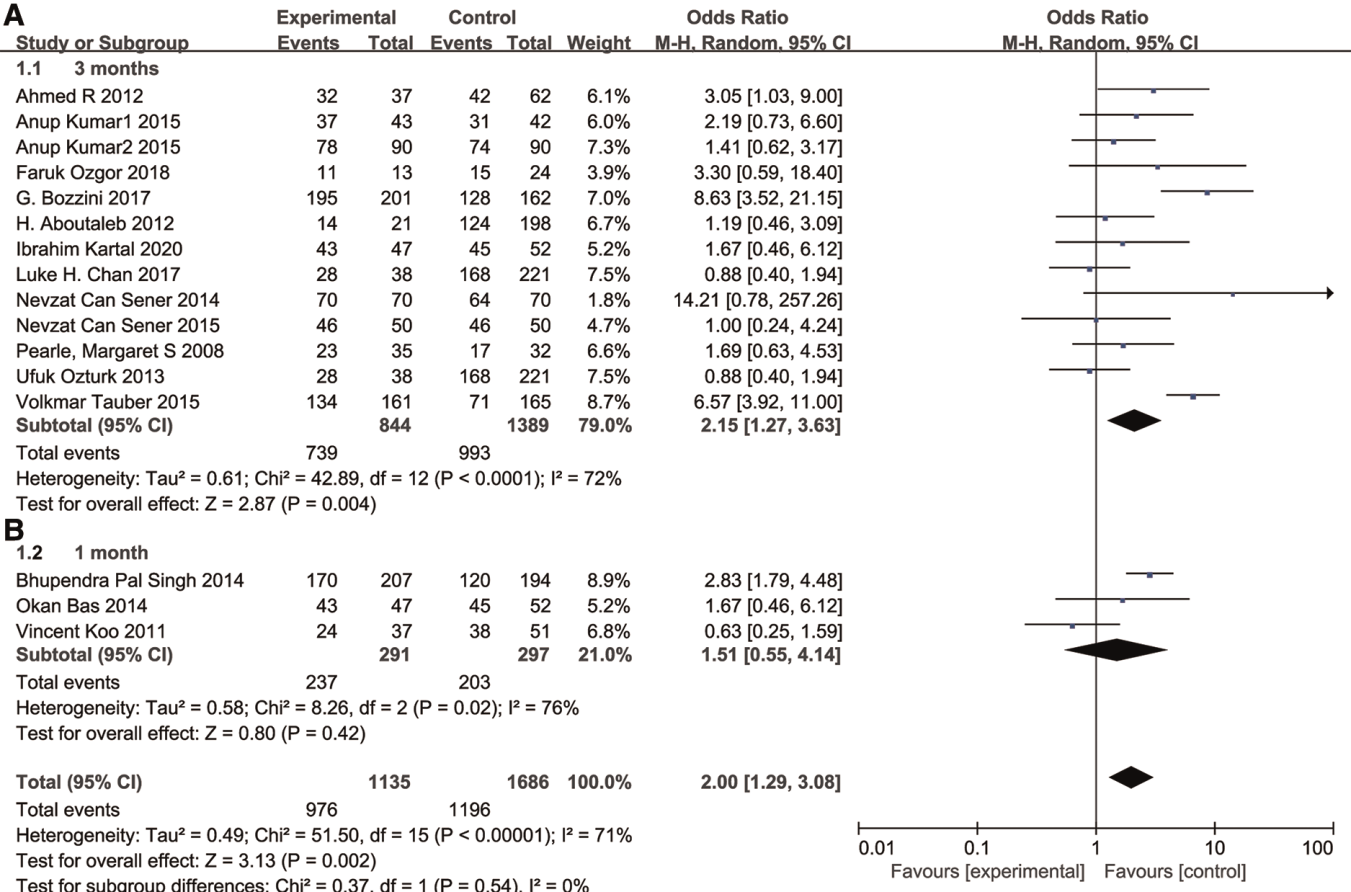
Forest plot and meta-analysis of stone-free rate for (**A**) 3 months after the surgery and (**B**) subgroups of the stone-free rate of 1 month after the surgery.

We also performed a subgroup analysis of SFR according to the cutoff time. After 3 months of the surgery, we found that SFR was higher in the f-URS group than that in the SWL group (OR: 2.15, 95% CI: 1.27–3.63, *p* = 0.004). However, no significant difference was found between the two treatment groups after 1 month of the surgery (OR: 1.51, 95% CI: 0.55–4.14), as shown in Figure 4.

#### Complication rate

**Figure 5:**
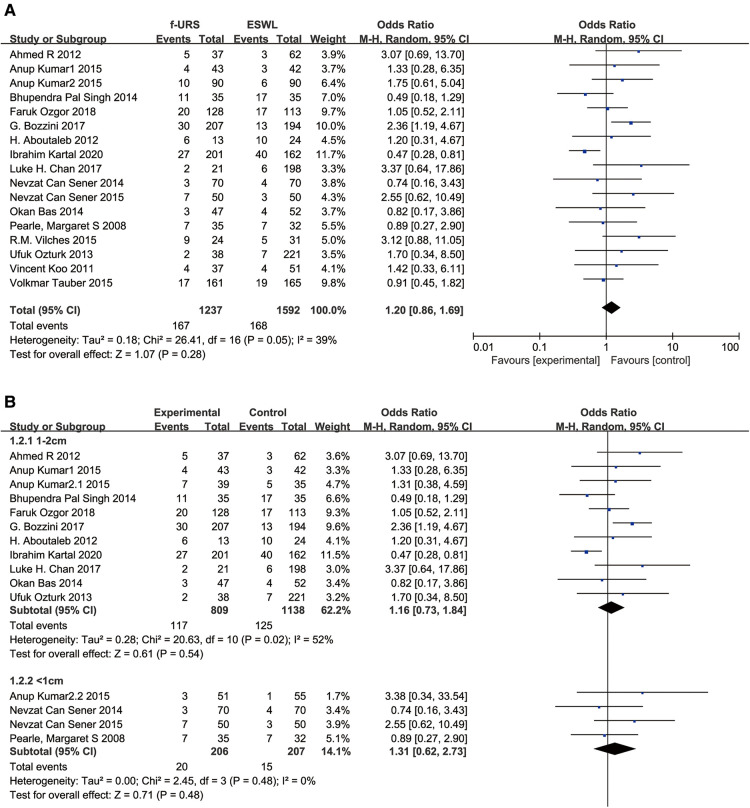
Forest plot and meta-analysis of complications rate for (**A**) all studies and (**B**) subgroups of stone length 1–2 cm and <1 cm for f-URS and SWL.

All 17 studies reported the complication rate in the f-URS group and SWL group. We found no significant difference between the two treatments in the complication rate (OR: 1.20, 95% CI: 0.86–1.69; *p* = 0.28), with moderate heterogeneity (*I*^2^ = 39%, *p* = 0.05). The same is true for the stone 1–2 cm subgroup (OR: 1.16, 95% CI: 0.73–1.84) and the stone <1 cm subgroup (OR: 1.31, 95% CI: 0.62–2.73), as shown in Figure 5. No publication bias was found using Egger's test (*p* = 0.060).

#### Retreatment rate

**Figure 6:**
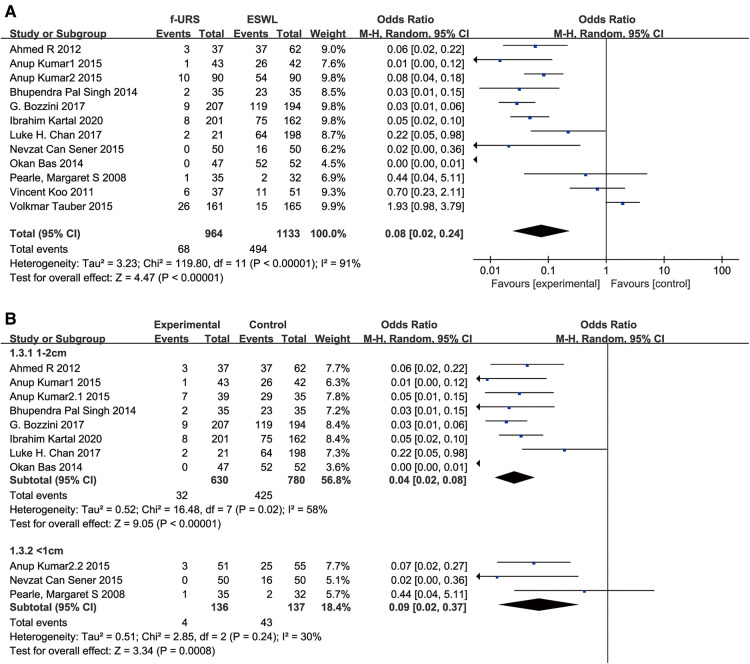
Forest plot and meta-analysis of retreatment rate for (**A**) all studies and (**B**) subgroups of stone length 1–2 cm and <1 cm for f-URS and SWL.

Twelve studies reported the retreatment rate in the f-URS group and SWL group. We found that the retreatment rate was significantly lower in the f-URS group than that in the SWL group (OR: 0.08, 95% CI: 0.02–0.24, *p* < 0.00001), with high heterogeneity (*I*^2^ = 91%, *p* < 0.00001). The same is true for the stone 1–2 cm subgroup (OR: 0.04, 95% CI: 0.02–0.08, *p* < 0.00001) and the stone <1 cm subgroup (OR: 0.09, 95% CI: 0.02–0.37, *p* = 0.0008). As shown in Figure 6, no publication bias was found using Egger's test (*p* = 0.321).

### Auxiliary procedure rate

**Figure 7:**
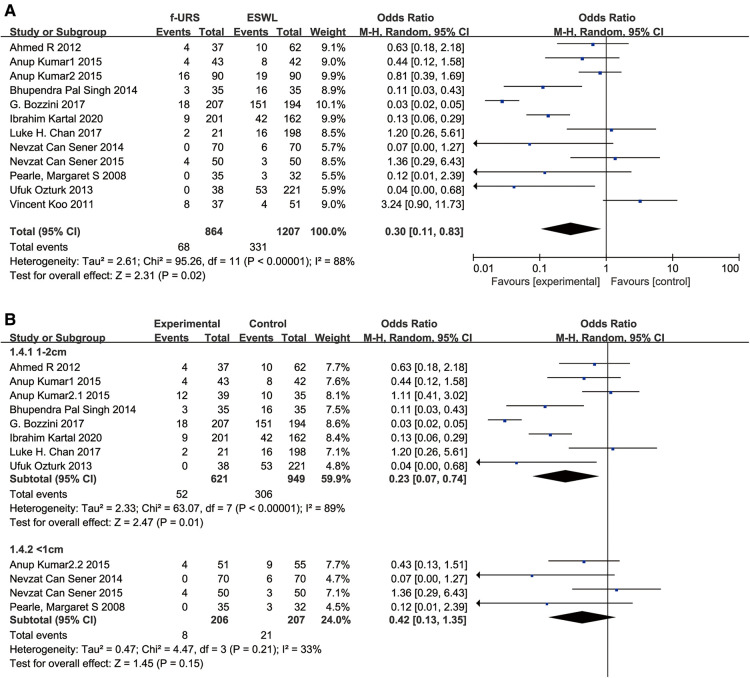
Forest plot and meta-analysis of auxiliary procedure rate for (**A**) all studies and (**B**) subgroups of stone length 1–2 cm and <1 cm for f-URS and SWL.

Twelve studies reported the auxiliary procedure rate in the f-URS group and SWL group. We found that the auxiliary procedure rate was significantly lower in the f-URS group than that in the SWL group (OR: 0.30, 95% CI: 0.11–0.83, *p* = 0.02), with high heterogeneity (*I*^2^ = 88%, *p* < 0.00001). The same is true for the stone of 1–2 cm subgroup (OR: 0.23, 95% CI: 0.07–0.74, *p* = 0.01). However, we did not find significant differences between the two groups in the stone <1 cm subgroup (OR: 0.42, 95% CI: 0.13–1.35, *p* = 0.15), as shown in Figure 7. No publication bias was found using Egger's test (*p* = 0.275).

### Operation time

**Figure 8:**
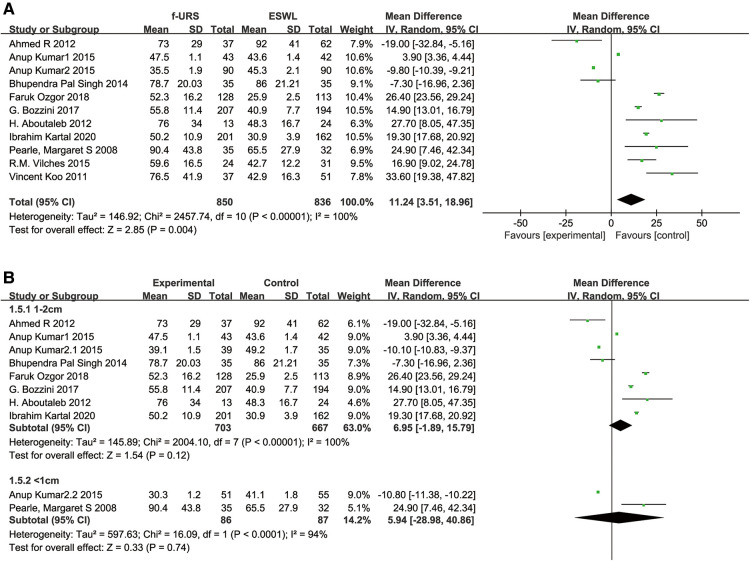
Forest plot and meta-analysis of operation time for (**A**) all studies and (**B**) subgroups of stone length 1–2 cm and <1 cm for f-URS and SWL.

Eleven studies reported operation time in the f-URS group and SWL group. We found that the operation time was significantly longer in the f-URS group than that in the SWL group (MD: 11.24, 95% CI: 3.51–18.96, *p* = 0.004), with significant heterogeneity (*I*^2^ = 100%, *p* < 0.00001). However, we did not find significant differences between the two groups in the stone <1 cm subgroup (MD: 6.95, 95% CI: −1.89 to 15.79, *p* = 0.12) and the stone 1–2 cm subgroup (MD: 5.94, 95% CI: −28.98 to 40.86, *p* = 0.74), as shown in Figure 8. No publication bias was found using Egger's test (*p* = 0.167).

### Hospital stay

**Figure 9:**
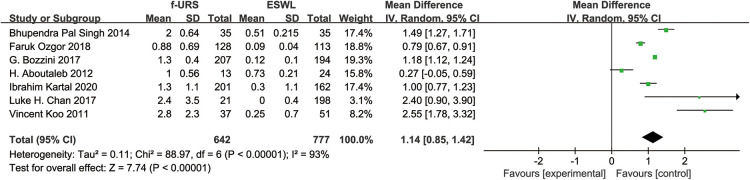
Forest plot and meta-analysis of hospital stay for f-URS and SWL.

Seven studies reported the length of hospitalization stay in the f-URS and the ESWL group. We found that the hospital stay was significantly longer in the F-URS group than that in the SWL group (MD: 1.14, 95% CI: 0.85–1.42, *p* < 0.00001), with a significant heterogeneity (*I*^2^ = 93%, *p* < 0.00001), as shown in Figure 9.

### Number of sessions

**Figure 10:**
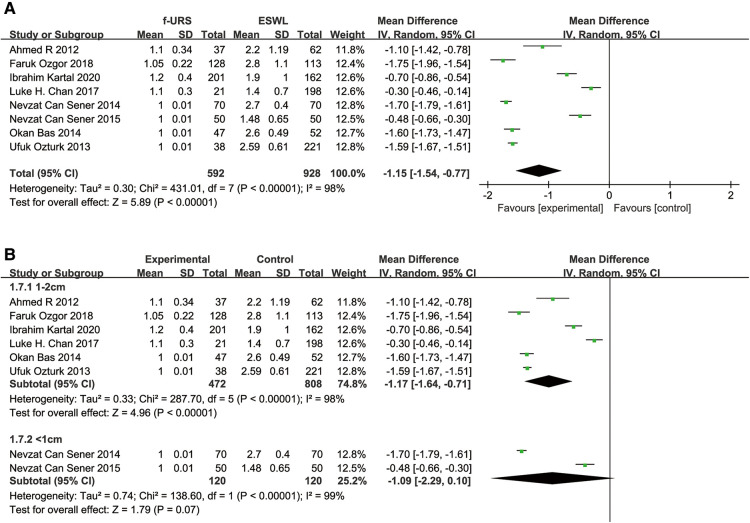
Forest plot and meta-analysis of the number of sessions for (**A**) all studies and (**B**) subgroups of stone length 1–2 cm and <1 cm for f-URS and SWL.

Eight studies reported the number of sessions in the f-URS group and SWL group. We found that the number of sessions in the f-URS group was significantly less than that in the SWL group (MD: −1.15, 95% CI: −1.54 to −0.77, *p* < 0.00001), with a significant heterogeneity (*I*^2^ = 98%, *p* < 0.00001), as shown in Figure 10. The same is true in the stone <1 cm subgroup (MD: −1.17, 95% CI: −1.64 to −0.71, *p* < 0.00001), but we did not find significant differences between the two groups in the stone 1–2 cm subgroup (MD: −1.09, 95% CI: −2.29 to 0.10, *p* = 0.07).

### Subgroup analysis

**Figure 11:**
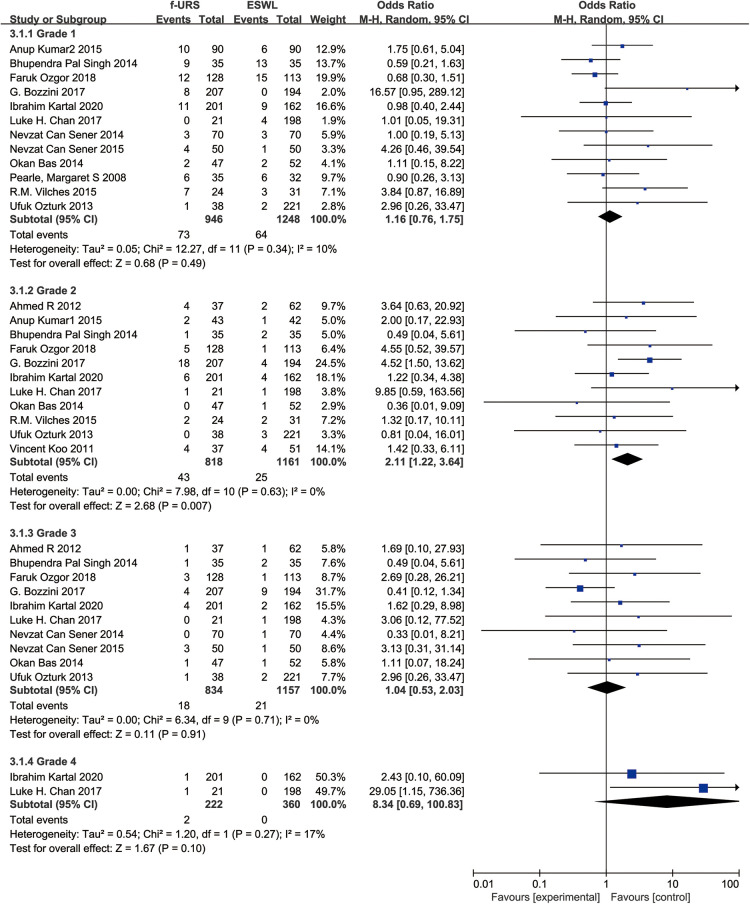
Subgroups analysis of complications graded by the Clavien–Dindo classification for f-URS and SWL.

To compare the incidence of complications of different grades between the two groups, we graded postoperative complications to grades 1, 2, 3, and 4 according to the Clavien–Dindo classification and performed a subgroup analysis (16). We found that there was no significant difference between the two groups in grade 1, 3, and 4 subgroups (OR: 1.16, 95% CI: 0.76–1.75; OR: 1.04, 95% CI: 0.53–2.03; OR: 8.34, 95% CI: 0.69–100.83), but the grade 2 complication rate in the f-URS was higher than that in the SWL group (OR: 2.11, 95% CI: 1.22–3.64), as shown in Figure 11.

### Sensitivity analysis

**Figure 12:**
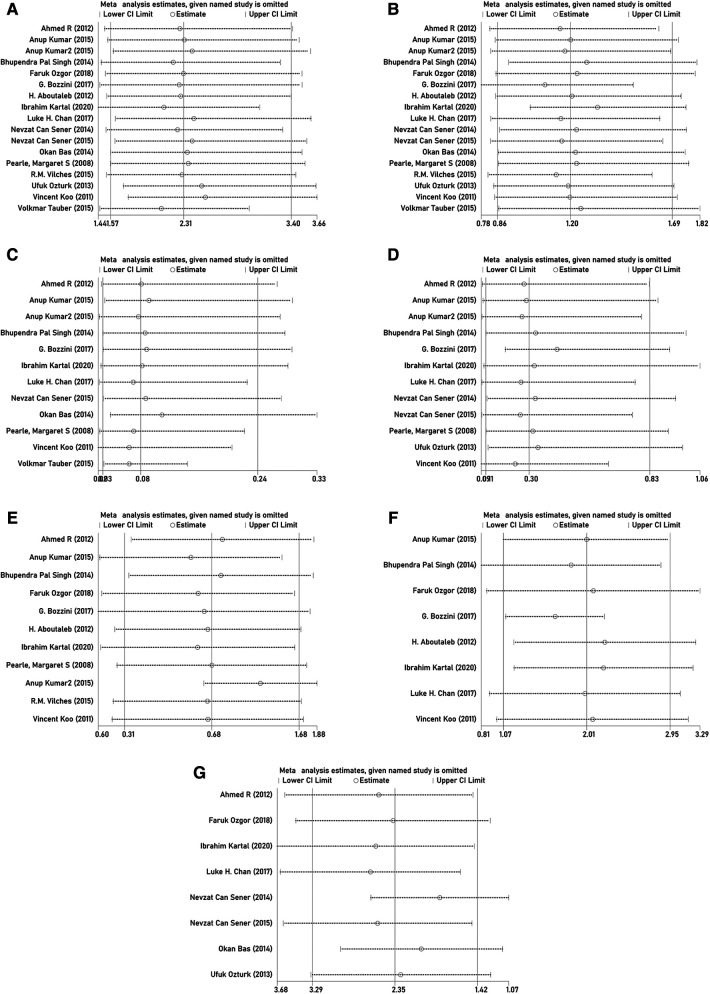
Sensitivity analysis of (**A**) stone-free rate; (**B**) complication rate; (**C**) retreatment rate; (**D**) auxiliary procedure rate; (**E**) operation time; (**F**) hospital stay; and (**G**) number of sessions.

Sensitivity analysis was conducted by omitting studies one by one. The pooled ORs based on the remaining studies in every group of meta-analysis were not out of the estimated range, as shown in Figure 12. No substantial variation was found between the adjusted and primary pooled estimates. Therefore, the strong robustness of our meta-analysis was confirmed.

## Discussion

The incidence of urolithiasis is increasing yearly worldwide, and its treatment methods and equipment are also constantly developing (32). The EAU guidelines point out that SWL and f-URS are optional treatments for nephrolithiasis ≤2 cm (33), and both treatments can achieve good SFR in the treatment of noninferior calyceal calculi ≤2 cm. In the management of ureteral stones, the SFR of SWL and f-URS is similar; however, it is easier to remove stones at one time with f-URS, but it has more complications (34).

Herout et al. found that, in Germany, although the incidence of urolithiasis has increased year by year and the number of surgeries to treat urolithiasis has also increased, the overall number of SWL has decreased and the proportion of SWL as an outpatient surgery has gradually increased. In contrast, the number of f-URS has increased significantly (35). The meta-analysis results of Mi et al. also found that for 1–2 cm urinary calculi, f-URS had higher SFR, lower auxiliary procedure rate, and lower retreatment rate than SWL (36). It seems that SWL has fallen behind f-URS in terms of safety and efficacy. However, the meta-analysis by Mi et al. was performed a long time ago, during which new studies on SWL and f-URS were published. Our work is to discuss the current safety and efficacy comparison between SWL and f-URS by collecting studies comparing SWL and f-URS in treating urolithiasis in recent years.

In our included studies, some provided more precise descriptions of stone length (the largest diameter of the stone), so we performed a subgroup analysis of these studies. Our study found that SWL compared with f-URS in the treatment of 1–2 cm stones had lower SFR, shorter hospitalization time, higher retreatment rate, higher number of sessions, and higher auxiliary procedures rate, which was similar to a previous study (36). However, in our subgroup analysis of <1 cm kidney stones, there was no significant difference in SFR between the two surgical modalities because when we extracted data from the study of Sener et al., its SFR was the statistical free rate after the same operation and the total clearance rate of other operations was added after the end of the study (27); this is the reason why our results were different from those of the study in 2016. There was no difference in operation time, complications rate, and the need for auxiliary procedures between SWL and f-URS. It shows that for kidney stones smaller than 1 cm, both SWL and f-URS can achieve similar SFR, and SWL may be a better choice because of its shorter hospitalization time and less impact on patients’ daily work and life (37).

The assessment of endpoint of SFR and the examined varied across studies. In most studies, the endpoint was three months after the surgery, and in Kumar et al.’s study, SFR was assessed 3 weeks after surgery using ultrasonography (22, 38). In the studies by Singh et al. and Bas et al., the SFR was assessed 1 month after each procedure (4, 29) and 2 months in the study by Vilches et al. Generally speaking, the longer the time from surgery, the easier it is for the residual stone to pass out, and the higher the SFR. When we extracted the data, the evaluation of SFR was performed after the same operation, which might include multiple sessions, but it was limited to the same operation and did not include other auxiliary operations. Different definitions of SFR and endpoint assessment among studies might have contributed to the biasedness. So, we performed a subgroup analysis according to the endpoint of SFR. After 3 months of the surgery, we found that SFR was higher in the F-URS group than that in the SWL group. However, no significant difference was found between the two treatment groups after 1 month of the surgery.

There was no statistical difference in the total complication rate of the two surgical procedures, and there was no statistical difference between the stone length subgroups, indicating that SWL and f-URS had similar complications in the treatment of ≤2 cm urinary calculi. After analyzing the complication classification, we found that SWL has fewer grade 2 complications than f-URS, which may be related to SWL, as, being a noninvasive procedure, it is less likely to cause complications such as urinary tract infection and sepsis; however, further research is needed to prove it. Notably, two studies reported two extremely severe grade 4 complications in patients receiving f-URS. Although serious complications of f-URS are rare with the development of technology and equipment, life-threatening complications still occur. SWL had fewer complications overall, but it was not statistically significant.

SWL showed a higher retreatment rate than f-URS, both overall and in subgroups, but with higher heterogeneity. The number of procedures associated with ESWL and the auxiliary procedure rate were also significantly higher than those of f-URS, which was similar to previous studies (33, 36). It showed that SWL needed to perform more times than f-URS to achieve full SFR.

The conclusions of each study showed great heterogeneity, which might be related to factors such as equipment used by different medical institutions in different countries and regions and the proficiency of technicians. There are many confounding factors, but, in general, most studies reported that SWL requires less operative time than f-URS, although operative time is defined by adding up the time spent in each session, which means that although SWL requires more treatments than f-URS, the overall time spent on surgery of SWL is still shorter than that of f-URS. A similar phenomenon occurred in the length of hospital stay, which is also the sum of the length of hospital stay for each operation. Although SWL costs more operations, the overall length of stay was still shorter than that of f-URS.

As more and more medical institutions perform SWL as an outpatient procedure, the advantages of SWL having less impact on the quality of life of patients are becoming much prominent (39). Although some medical institutions have also begun to try to perform f-URS as an outpatient procedure, characteristics of f-URS as an invasive procedure requiring anesthetic drugs limit its development (40). The evaluation of the impact of the two surgical methods on the quality of life of patients may become the next focus. Although some studies have reported the impact of the two surgical methods on the quality of life of patients in recent years (37, 39, 41), the forms and methods of questionnaires used by the research institutes were quite different and the conclusions they drew were also different. At present, a unified, standardized, and quantifiable method is urgently needed to evaluate the two surgical methods, and further research is needed for evaluation in the future.

This study still has some limitations. First, as noted above, studies have inconsistent cutoff times for SFR assessments and different definitions of SFR, which may create a range of biases. Second, there are few studies comparing the safety and efficacy of SWL and f-URS in recent years, so the included literature has not been significantly improved in quantity and quality compared with the previous meta-analysis. Finally, the differences in medical technology and systems in various countries and regions will also cause certain biases accordingly.

## Conclusion

For 1–2 cm urinary stones, f-URS can achieve a higher SFR than SWL while having a lower retreatment rate, number of sessions, and auxiliary procedure rate. For urinary stones <1 cm, there is no discernible difference in SFR between SWL and f-URS. While SWL is mostly performed as an outpatient procedure, f-URS mostly requires hospitalization. SWL has a shorter total operative time and total hospital stay than f-URS, regardless of the length of urinary stone treatment. After 1 month of the surgery, the SFR between f-URS and SWL showed no difference. However, after 3 months, f-URS showed higher SFR than SWL.

## Data Availability

The raw data supporting the conclusions of this article will be made available by the authors, without undue reservation.
