# Supplementary figures and images for: Safety and efficacy of extracorporeal shock wave lithotripsy vs. flexible ureteroscopy in the treatment of urinary calculi: A systematic review and meta-analysis
Source: Front Surg. 2022 Nov 7;9:925481. doi: 10.3389/fsurg.2022.925481 (PMC9676362; doi:10.3389/fsurg.2022.925481)

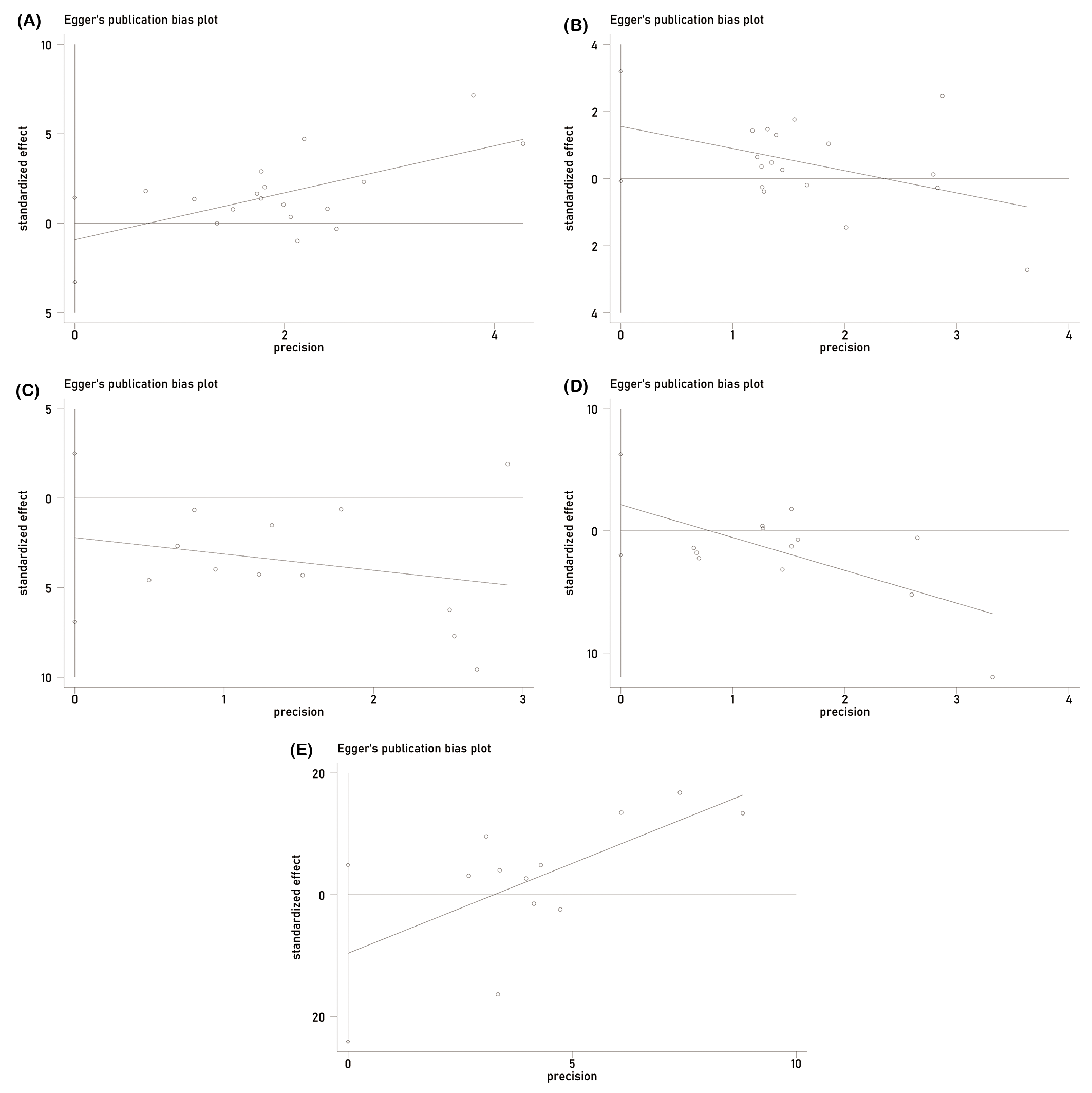

Supplement: Supplementary file 1 [file Image1.tif]
